# Supplementary material for: Lin28a/let-7 pathway modulates the Hox code via Polycomb regulation during axial patterning in vertebrates
Source: eLife. 2020 May 29;9:e53608. doi: 10.7554/eLife.53608 (PMC7259951; doi:10.7554/eLife.53608)
Supplement: Supplementary file 1. [file elife-53608-supp1.docx]

**Supplemental Table 1. Survival rate of *Lin28a* mutant mice at various stages.**

|  | | Number of pups | | | % of each genotype | | |
| --- | --- | --- | --- | --- | --- | --- | --- |
| Stage | Litters | Wt | *Lin28a^+/–^* | *Lin28a*^–/–^ | Wt | *Lin28a^+/–^* | *Lin28a*^–/–^ |
| E9.5–13.5 | 15 | 42 | 59 (1) | 23 (3) | 35% | 48.3% | 16.7% |
| E15.5 | 5 | 12 | 18 | 11 | 29.3% | 43.9% | 26.8% |
| E17.5 | 5 | 12 | 14 | 4 (1) | 41.4% | 48.3% | 10.3% |
| Neonate | 30 | 75 | 131 | 14 (3) | 34.6% | 60.4% | 5.1% |
| Adult | 28 | 63 | 116 | 4 | 34.4% | 63.4% | 2.2% |

The number of dead offspring and embryos is shown in parenthesis.
